# Critical Review on Polylactic Acid: Properties, Structure, Processing, Biocomposites, and Nanocomposites

**DOI:** 10.3390/ma15124312

**Published:** 2022-06-17

**Authors:** Lalit Ranakoti, Brijesh Gangil, Sandip Kumar Mishra, Tej Singh, Shubham Sharma, R.A. Ilyas, Samah El-Khatib

**Affiliations:** 1 Department of Mechanical Engineering, Graphic Era Deemed to be University, Dehradun 248002, India; lalit_9000@yahoo.com; 2 Mechanical Engineering Department, SOET, HNB Garhwal University, Srinagar 246174, India; brijeshgangil@gmail.com; 3 Mechanical Engineering Department, IET, Bundelkhand University, Jhansi 284128, India; sandipmishra003@gmail.com; 4 Savaria Institute of Technology, Eötvös Loránd University, 9700 Szombathely, Hungary; tejschauhan@gmail.com; 5 Mechanical Engineering Department, University Center for Research & Development, Chandigarh University, Mohali 140413, India; 6 Department of Mechanical Engineering, Main Campus, IK Gujral Punjab Technical University, Kapurthala 144603, India; 7 Faculty of Engineering, School of Chemical and Energy Engineering, Universiti Teknologi Malaysia, Johor Bahru 81310, Malaysia; ahmadilyas@utm.my; 8 Centre for Advanced Composite Materials, Universiti Teknologi Malaysia, Johor Bahru 81310, Malaysia; 9 Mechanical Engineering Department, Faculty of Engineering and Technology, Future University in Egypt, New Cairo 11835, Egypt

**Keywords:** polylactic acid, biodegradability, processing, biocomposites, nanocomposites

## Abstract

Composite materials are emerging as a vital entity for the sustainable development of both humans and the environment. Polylactic acid (PLA) has been recognized as a potential polymer candidate with attractive characteristics for applications in both the engineering and medical sectors. Hence, the present article throws lights on the essential physical and mechanical properties of PLA that can be beneficial for the development of composites, biocomposites, films, porous gels, and so on. The article discusses various processes that can be utilized in the fabrication of PLA-based composites. In a later section, we have a detailed discourse on the various composites and nanocomposites-based PLA along with the properties’ comparisons, discussing our investigation on the effects of various fibers, fillers, and nanofillers on the mechanical, thermal, and wear properties of PLA. Lastly, the various applications in which PLA is used extensively are discussed in detail.

## 1. Introduction

Relentless exploitation of synthetic materials to dispense necessary comforts to the user has intensified the state of deterioration of the environment and has put the world at an alarming need for the immediate replacement of man-made materials with naturally occurring substances [1,2,3]. Various commodities such as drinking bottles, packaging materials, electronic gadgets, toys for children, and other innumerable item used daily are being manufactured from the petroleum-based material called plastic [4,5]. The dire need to replace plastic with a biopolymer is felt because petrochemicals require a large amount of energy during processing and release CO_2_ that has detrimental effects on the environment [6]. In addition, the use of plastic produces nonbiodegradable waste and depreciates the fertility of soil. On the other hand, biopolymers can be processed using comparatively low energy, and the waste produced can be easily disposed of [7]. Although reusing and recycling plastics have become a common practice in various countries and industries to reduce the accumulation of polymer waste, it cannot be afforded by developing and underdeveloped countries because of the high cost associated with it [8,9]. Therefore, various measures have been taken to curtail the ensuing effects of the use of polymers. The most familiar and effective measure is the replacement of polymers with biopolymers. Biopolymers are obtained naturally. Moreover, the degradation is made more accessible by microorganisms in a short time. Biopolymers are classified into three groups, viz., those (1) synthesized from monomers, (2) produced by microbial fermentation, and (3) naturally modified, as mentioned in Table 1.

According to a survey carried out in 2020 for market research, the total market capitalization of polymer and biopolymer industries was estimated to be USD 666.6 billion and USD 10 billion, respectively, and is expected to grow at the rate of 16% per year [13]. An increase in the volume of polymer waste was also observed and found to be 320 million tons in 2020, as reported in the yearly statistics given by the United Nations [14]. In order to reduce the capital share of nonbiodegradable polymers in the market, several countries (mainly developed countries) have come together to promote biopolymers in several ways. Statistics suggested that Europe alone contributed to 55% of the total volume of biopolymers produced in 2018, mostly restricted to such industries as packaging, compost bags, food and beverages, and fabrics [15]. Nonetheless, more efforts are being made to increase the production of biopolymers and to expand their scope to various segments; if we fail to do what is necessary, the time will not be far off when we shall see the present world turn into a place in which the survival of humankind would be worrisome.

Due to technological advancement and research, biopolymers can play a vital role by gradually replacing plastic in various sectors, such as the biomedical, agriculture, household utility industries, etc. [16,17,18]. Although the share of biopolymers in the market is relatively small, the rate at which it is increasing is appreciable. However, the market capitalization of biopolymers is less than that of polymers because various factors, such as complications in processability, higher per-unit cost due to low production, comparable low mechanical strength, dissimilarities in resources, etc., restrict their widespread utilization [19]. Nevertheless, research and development groups are working continuously to address these issues, as the co-blending of polymers and the use of plasticizers can improve the processability and strength of biopolymers [20,21,22].

Furthermore, carrying out socioeconomic programs to promote awareness of the detrimental effects caused by polymer products will help expand the scope of biopolymers in the sphere of polymeric composites. Additionally, the soaring price of fossil fuel indicates that, soon, it is expected that biopolymers will be preferred over plastic to develop cheap and sustainable products used in daily life [23].

**Figure 1:**
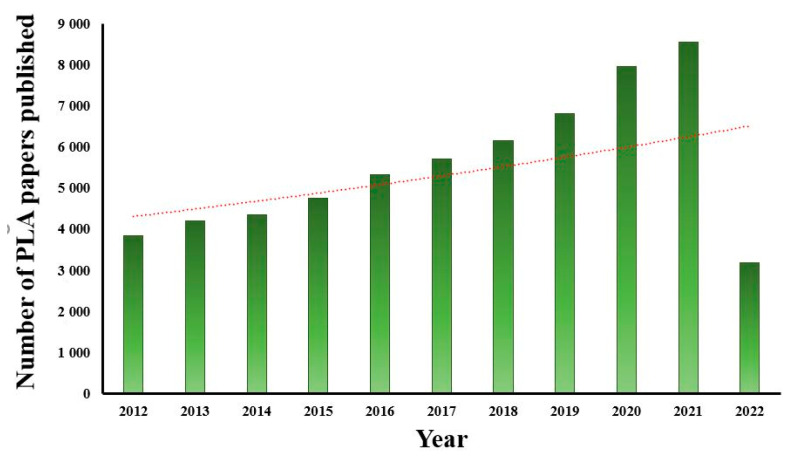
Number of papers published in PLA-based composites (through April 2022) (data collected from Web of Science).

Biocomposites are now receiving more attention in polymer composites’ materials because of the inherent characteristics of biodegradability [24]. Additionally, the number of papers published on PLA has witnessed an increasing trend in the last 10 years, as shown in Figure 1, demonstrating the importance of PLA in the field of composites. The reason behind a smaller number of papers being published in 2022 is because papers were counted through the month of April. A huge amount of biopolymers is extensively used in the manufacturing of biocomposites. These biocomposites are fabricated by the combination of various biopolymers and biofibers. Various types of biopolymers have been used as a matrix material with different reinforcements to fabricate biocomposites. PLA is mainly preferred among biopolymers due to its adequate compatibility with various natural reinforcements and comparable mechanical and physical properties [25]. It is a lactic acid formed by the fermentation of carbohydrates (found in corn, which is a cheap source) carried out by bacteria using the Lactobacillus genus (an optimized strain). It is economically feasible and can be produced at the cost of USD 0.25/pound [26]. Polylactic acid has several advantages, e.g., it is a source of agriculture-based products, helps fix the substantial amount of CO_2_ (a greenhouse gas), requires less energy in processing, is easily recycled into lactic acid without much effort, and has tailored physical–mechanical properties through material modifications [27]. Hence, a comprehensive and informative review article is necessary to address all the important issues comprising the properties of PLA and its significance; its composites fabricated via various processes by incorporating different fibers, fillers, and nanofillers; their respective characterization; and, finally, its scope in various industries.

## 2. Basel Convention

As per the Basel Convention, the production and use of hazardous polymers consisting of organic polymers are strictly prohibited. These polymers are comprised of hazardous organic pollutants that require efficient waste management. The polymers that fall under this category are styrene copolymers, polyacrylonitriles, polyvinyl chloride, epoxy resins, and polyurethanes. Various chemicals are added to these polymers to confer certain properties such as flame retardancy. Article 16 of the convention deals with the technical assistance and capacity building steps to manage plastic waste. Uncontrolled burning and disposal of plastic waste into the marine environment are severe issues that cannot be resolved alone by waste management and, hence, require immediate replacement by biodegradable plastic such as PLA. The Basel Convention for hazardous plastic waste (B3011) amendment deals with the recycling of plastic waste in an eco-friendly manner, thus free from any hazardous waste. Later, insertion into the Basel Convention for plastic waste (Y48), which became effective on 1 January 2021, included the listed mixture of plastic wastes, which are nonhazardous and fall under the category of B3011. The Stockholm Convention on Persistent Organic Pollutants states that compatibilizers such as retardants and plasticizers are added to synthesize polymers to regulate properties such as flexibility, transparency, and durability. These substances are organic pollutants that need to be reformed [28].

Solid plastic waste was listed as a nonhazardous material before the 2019 amendment in the Basel Convention. However, post amendment, all the plastic wastes are in the hazardous category. However, the Basel parties are not legally obliged to adopt the terms in the amendment. The answer to this ambiguity is the use of biodegradable polymers, such as PLA. Since it has shown compliance with the majority of the convention, as proposed in Basel, its usage in the larger scale would be beneficial in terms of both the environment and economy.

## 3. Structure and Properties of Polylactic Acid

Due to the similarities of several properties of PLA with various polymers, as illustrated in Table 2, such as polyvinyl chloride, polystyrene, etc., it is considered one of the fastest emerging biopolymers in the arena of composite materials [28]. A property comparison of PLA with typical polymers reveals that PLA has a higher tensile modulus than PVC, PP, and nylon while it has a higher flexural strength than PP, indicating the potential in PLA, which can be explored in various ways in engineering domains. PLA has an immense potential for improvement in its physical and mechanical properties, which was reported in the earlier literature of the last two decades [29]. In addition, PLA can be processed by a simple conventional process without using a great deal of energy or time inputs, thus making it an inexpensive and readily available polymer [30,31,32]. Furthermore, it can be easily molded and reshaped in a large number of forms through various processes, such as extrusion and injection molding [33,34,35,36,37,38,39,40], for which it is gaining more attention in food industries for replacing the leading polymers shortly, as listed in Table 2.

The physical observation of PLA has its significance in the prospect of industrial applications. It is shiny and transparent, remains stable at low temperatures, has modest oxygen and water permeability, and has high grease and oil resistance. These properties make it suitable for manufacturing film, bottles, cups, and trays [36]. However, the properties listed in Table 3 do not remain constant but vary with D-content and molecular weight changes. For instance, on increasing the molecular weight of PLA, the crystallinity decreases due to the forming of a long polymer chain. However, both tensile strength and shear viscosity increase due to chain entanglement and length [37]. A unique characteristic, known by the term variable stereochemistry, can be applied to PLA, bringing significant changes in the properties, leading to further possibilities of tailoring in the properties, which can be considered an advantage of PLA over other petrochemical polymers. Stereochemistry causes different chemical structures in the PLA, resulting in semicrystalline and amorphous structures depending on the ratio of the D- and L-content [38]. The physical and mechanical properties and the rate of biodegradation depend on the chemical structure of PLA. The D-content is an important parameter, enabling the alternation of the properties of PLA in several ways; an increase in the D-content in PLA can lead to a decrease in the rate of crystallization, which, in turn, results in the lowering of the melting point [39]. Plasticization of PLA can also be carried out to improve its crystallinity by blending with other polymers such as starch, citrate ester, polyethylene glycol, sorbitol, and oligomeric lactic acid [40].

**Figure 2:**
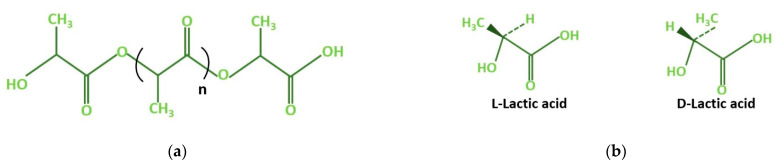
(**a**) Chemical structure of polylactic acid; (**b**) isomers of lactic acid.

Polylactic acid belongs to the family of poly-α-hydroxy acid, a type of linear aliphatic thermoplastic polyester, as shown in Figure 2a. The enantiomeric form of PLA is divided into three categories, viz., levorotatory (L-), dextrorotatory (D-), and meso (a combination of L- and D-), as shown in Figure 2b [41]. The type of PLA formed will depend on the source form and the process followed in making it. A subclass of meso, called “racemic mixture”, can also be formed if the ratio of L- and D- present in the mixture is equal. The polymer formation using L-, D-, and meso enantiomers is termed poly L lactic, poly D lactic, and poly meso lactic, respectively [42].

## 4. Production of PLA

**Figure 3:**
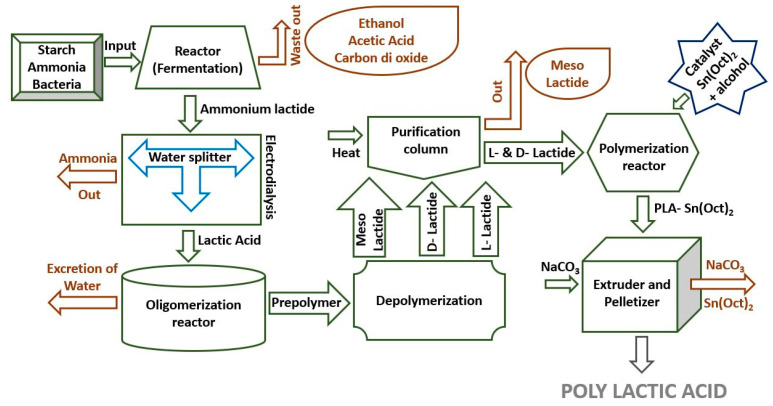
Process of making polylactic acid.

Production of lactic acid can be executed either biologically or chemically with the help of bacteria. The judicious selection of the bacteria can control the development of enantiomers during the process. Bacteria expedites the reaction by breaking the compounds in L-lactic and D-lactic acid, consequently reducing the molecular weight of the polymer [43]. A detailed explanation of PLA production from an agricultural product is shown in Figure 3. The process initiates with the fermentation of starch in ammonia and bacteria in a reactor that produces ammonium lactate and separates acetic acid, carbon dioxide, and alcohol. Ammonium lactate then travels to water-splitting chambers where ammonia is separated from ammonium lactate by electrodialysis. This process uses the electro potential of cation and anion to separate ammonium lactate and produces lactic acid. The lactic acid is then fed to an oligomerization reactor where water is excreted and lactic acid changes into a prepolymer. The prepolymer coming out of the oligomerization reactor enters into a depolymerization reactor where each monomer from the free polymer is broken down into its sub-units and forms rings of lactide, essentially required for the ring-opening process. This form different stereo isomers of lactide called L-lactide, D-lactide, and meso lactide [44]. The three stereo isomers are fed to the purification column where, with the help of heat, meso lactide in the form of liquid is separated from L- and D-lactides. After separating meso lactide, L- and D-lactides are fed to a polymerization reactor where ring-opening occurs. A metal catalyst is needed to carry out the ring-opening process.

Numerous catalysts can be used, e.g., zinc, titanium, and tin. Tin is the most well-known catalyst used for polymerizing lactides, and it is used in the form of stannous octoate (Sn(Oct)_2_). Stannous octoate is added with alcohol and forms a more complex molecule that allows the ester oxygen to attach when it reacts with lactide. This will cause the oxygen to lose its double bond and open up into a polylactic acid chain. Stannous octoate opens many lactide rings, forming a long chain of PLA. A carboxylic acid is used with Sn(Oct)_2_ as a catalyst as an alternate method instead of using alcohol. However, it is not often used because the amount of PLA produced is much less than the amount produced by alcohol. Although PLA is produced in the polymerization reactor, the Sn(Oct)_2_ is still attached to the PLA, which is eliminated in the extruder and pelletizer chamber [45]. The sodium carbonate is added as a catalyst, which reacts with Sn(Oct)_2_ and gets separated through a string. The newly formed pure PLA is in a liquid form, solidified by using a drier, and transformed into desired shapes. Apart from that, various other processes such as ring-opening polymerization, condensation polymerization, or azeotropic fermentation can also be used for the mass production of PLA; but the process, as shown in Figure 3, is a flexible process in which D, L, and meso compounds can be extracted at the same time. Moreover, a high yield of PLA can also be achieved by changing the catalyst.

## 5. Biodegradation of PLA

**Figure 4:**
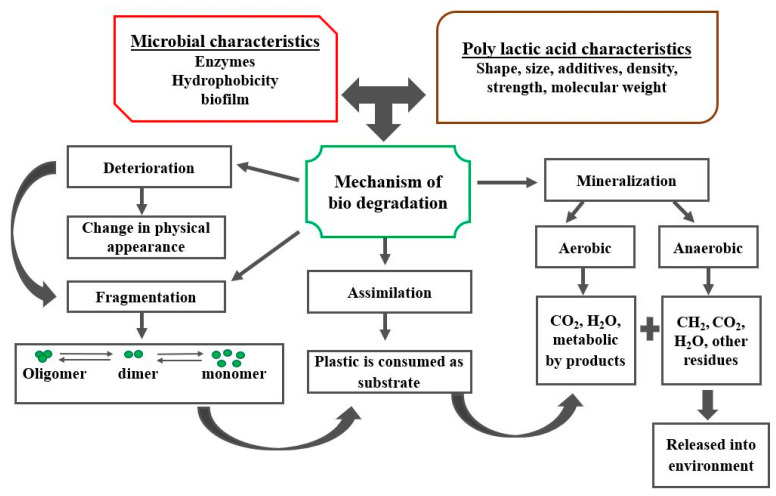
Biodegradation mechanism of PLA.

Biodegradation of PLA takes place naturally in the presence of algae, fungi, and bacteria. The chemical structure of the PLA breaks into H_2_O, CO_2_, and inorganic compounds, as shown in Figure 4. Meanwhile, some amount of biomass is also produced during the process, which can be utilized as manure. The term biodegradability becomes identical to compostability when the residue obtained is nonvisible and nonpoisonous [46]. Biodegradability is only ensured under a specific set of conditions governed by temperature, pressure, and humidity. Unfavorable environmental conditions will slow down the rate of biodegradation, which may differ from the natural biodegradation in terms of the obtained residue even in the presence of enzymes. Biodegradation of PLA occurs in two phases, heterogenous (surface degradation) and homogenous (intramolecular degradation). The degradation of any polymer takes place in three ways: (1) scission through an intersectional chain, (2) scission through the main chain, and (3) scission through a side chain. For PLA, biodegradation transpires through the emission of ester bonds, breaking down the long polymer chain into short oligomers, monomers, and dimers precisely known as carboxylic acid and alcohol. These short units can penetrate through the cell wall of microorganisms, leading to their biochemical degradation. Factors influencing the biodegradation of PLA are melting point temperature, glass transition temperature, crystal structure, molecular weight, chemical affinity, surface area, etc. For instance, PLA with higher molecular weight degrades slowly compared to PLA with lower molecular weight. The higher the melting point of PLA is, the lower will be its rate of degradability [47].

The structure of PLA also has a significant effect on the rate of biodegradability since the amorphous structure is more prone to degradation than the crystalline structure. In this regard, an investigation was carried out to examine the degradation rate of amorphous and crystalline PLA by keeping it in a phosphate-buffered solution of pH 4.0 at 37 °C. Findings revealed that amorphous PLA degraded 14% in 4 months while an equal degradation in crystalline PLA took place in 20 months [48]. PLA can be degraded both in the presence and absence of oxygen, i.e., in aerobic and anaerobic environments, respectively. The degradation phenomena of PLA in an aerobic environment have been extensively studied, whereas only a few researchers have examined the degradation behavior of PLA in an anaerobic environment. It was observed that the PLA degrades slowly in an anaerobic environment. However, increasing the degrading temperature above the glass transition T_g_ of PLA can accelerate the degradation rate. Aerobic degradation is similar to compositing under the action of various micro-organisms where the product of decomposition is humus [49]. Composting is mainly used to degrade industrial waste, where a large amount of waste is collected every day. A comparative study was carried out to examine the degradation rate of PLA at home and in industry. It was reported that the PLA degraded at a very fast rate (60–80 days) in industrial composting compared to home compositing because of the influence of several factors, such as high temperature, high pressure, and suitable humidification [50]. Due to the presence of a hydrolyzable functional group in PLA, it is vulnerable to hydrolysis.

The biodegradation of PLA in aerobic conditions transpires in two stages. In the first stage, water diffuses in the hydrolysis chain; in the second stage, the micro-organisms attack the weak molecular chain and convert it into CO_2_, H_2_O, and microbial biomass. It is worth noting that an enzymatic attack on the PLA can only happen when the molecular weight falls to 10,000 Da or less. Various enzymes vital in the fast degradation of PLA are lipase, cutinase, alkaline proteases, subtilisin, trypsin, and elastase [51]. These enzymes are readily found in algae, fungi, and bacterial bodies.

## 6. Fabrication of PLA Composites for Engineering Applications

There are several ways of producing fibers/fillers-based PLA composites.

**Figure 5:**
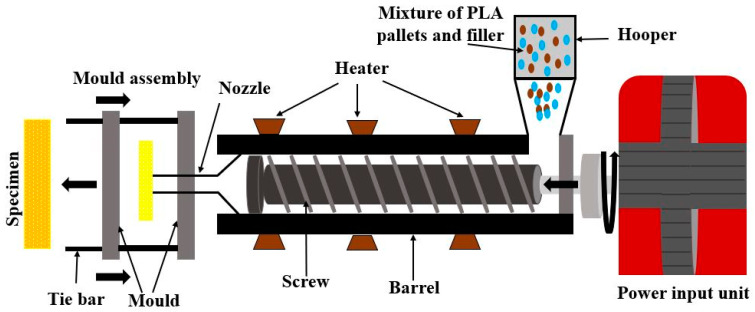
Twin-screw extruder for the fabrication of fillers-based PLA composites.

(a) Twin-screw extruder: It is generally equipped with a separate hooper in which pallets of PLA and fillers are mixed, as shown in Figure 5. The mixture is then fed to the horizontal barrel, which is rotated by centrifugal force provided by an external power source. Meanwhile, the mixture is heated stepwise to convert the pallets of PLA into a liquid form. A liquid mixture of PLA and filler is passed through a converging nozzle to minimize the chances of air entrapment in the solution [52]. Finally, the slurry of the mixture is forced into the mold to fabricate the required shape of the specimen.

Mixing compatibilizers, stabilizers, and solvents is encouraged as a homogenous solution is easily achieved due to the rotation. Uniform dispersion of fillers in the PLA matrix is difficult to achieve as light-density filler particles get segregated at the center of the extruder [53]. The shape of the twin-screw extruder does not allow the reinforcement of fiber mat in PLA; hence, only filler reinforcement is added in this process.

**Figure 6:**
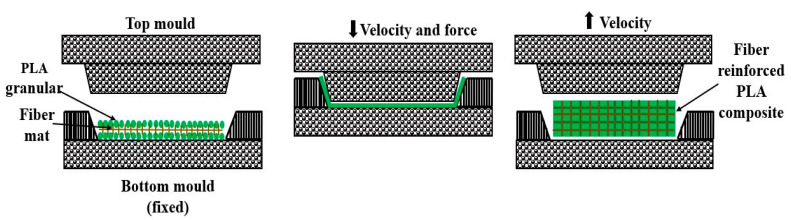
Compression-molding method.

**Figure 7:**
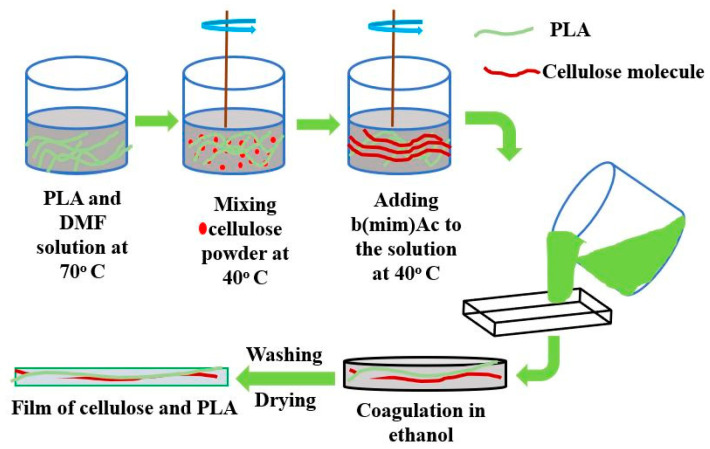
Preparation of cellulose–PLA film.

**Figure 8:**
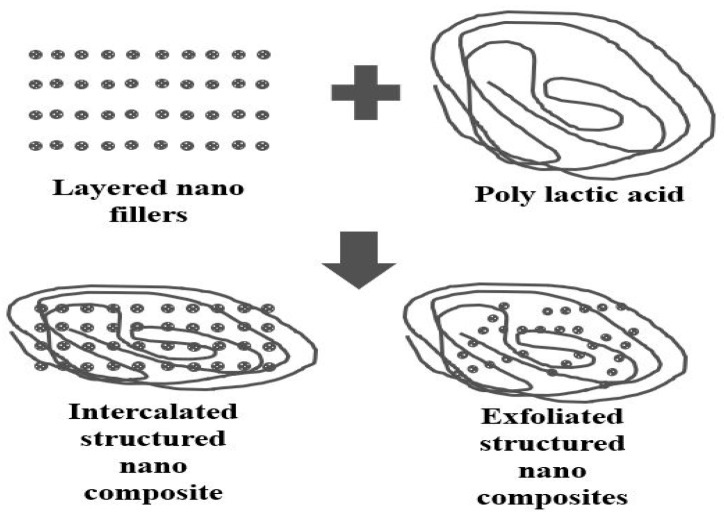
Intercalated and exfoliated structures.

(b) Compression molding technique: It is common to make fibers-reinforced polymer composites in which reinforcement is supplied in the form of a fibers’ mat, as shown in Figure 6. The fibers’ mat and pallets of PLA are placed in the bottom mold, which is fixed, and are heated to the melting temperature of PLA. Upon melting, the PLA spreads uniformly over the surface of the fiber mat. The mixture is then pressed between the two molds at a pressure of 20 KPa by bringing down the top mold through hydraulic means. After some time, the molds are separated, and the composites are taken out [54]. Filler-reinforced PLA composites are difficult to fabricate by this process due to their nonhomogeneity [55].

(c) Cellulose-PLA film: Over the past years, several methods have evolved for the fabrication of PLA films, for example, the incorporation of cellulose fibrils in the PLA as an additive, layer by layer deposition or coating of cellulose on PLA, acetyl nitrate-treated modified cellulose–PLA films, and film produced by a hot press method [56]. However, polylactic acid films fabricated by the methods above encounter several problems such as heterogeneity, complexity in mechanism, and time consumption. Additionally, the film produced is brittle and weak in strength. A novel method, known as an ionic liquid/co-solvent system, was recently introduced to overcome these problems, as revealed in Figure 7. The procedure follows mixing PLA dimethylfuran (DMF) at 70 °C to prepare a solution. The temperature of the solution is then lowered by stirring, and cellulose microfibrils are added. After some time, 1-Butyl-3-methylimidazolium acetate [bmim]Ac is added to the solution. The stirring is continued until the cellulose dissolves completely in PLA/DMF/[bmim]Ac. The solution is then coagulated in ethanol and, subsequently, after washing and drying, fabricated as the film of cellulose–PLA [57].

(d) In situ polymerization: Polylactic acid nanocomposites are prepared by mixing with ceramic nanofillers having a layer. Next, the catalyst is added to the mixture and subjected to ring-opening polymerization [58]. The nanocomposites thus produced can exhibit layered or exfoliated structures, as shown in Figure 8.

The dispersion of silicates’ nanofiller can be controlled by treating the nanofiller with a suitable chemical agent. In the process of solvent intercalation for the synthesis of PLA nanocomposites [59], the solvent dissolves the PLA for easy dispersion of nanofillers. The mixture of nanofiller and PLA is stirred and subjected to high-speed sonication. In melt intercalation, the heating of ceramic nanofillers above the glass transition temperature is carried out. The blending of nanofillers with PLA is then performed using an extruder under the action of shear force [60]. The improved interaction of the layered silicates with PLA is achieved by surface modification of the nanofiller. The processes discussed above for the fabrication of PLA-based materials have their respective merits and demerits; for example, melt extruder has the capability of producing filler–PLA composites at a higher rate of productivity, whereas compression molding can produce high-strength, fiber-reinforced PLA composites but it is a time-consuming process. Film based on PLA prepared by the solvent technique improves the mechanical properties and induces toughness, hence widening its application.

## 7. Effect of Natural Fibers/Fillers on the Properties of PLA Biocomposites

**Figure 9:**
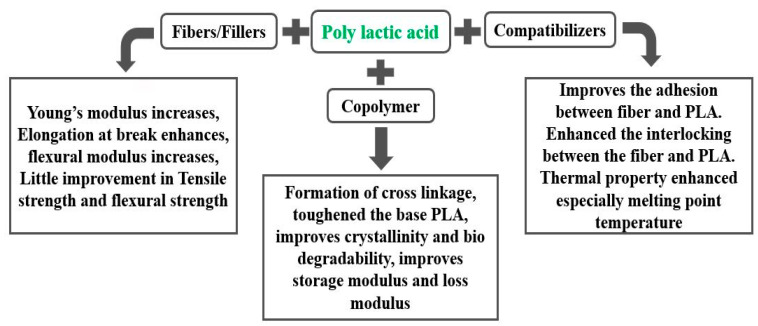
Summarized effect of various reinforcements in PLA.

The quest for producing a fully biodegradable composite with reasonable mechanical properties seems to end with the progress of PLA biocomposites. In order to investigate the potential of PLA as a polymer matrix, numerous biocomposites were fabricated with the help of various manufacturing methods. In an effort to fabricate biocomposites, sugar beet pulp of 7 to 40 wt.% was mixed with PLA in an injection molding machine [61]. Results showed that Young’s modulus of PLA increased by 45%, whereas tensile strength decreased by 29% due to the addition of the sugar beet pulp. The unfavorable result was attributed to the length of the short fiber leading to an inefficient stress transfer mechanism between the fibers and matrix. The mechanical properties of the PLA decreased by 50% when reinforced with oil seed fibers [62]. Improper distribution of reinforcement due to the inept manufacturing method resulted in a reduction in the mechanical properties of PLA. However, changing the process of fabrication can enhance the mechanical properties. Mixing aluminum triflate with PLA in a blender followed by microwave heating at 170 °C for 6 h [63] would produce biocomposites exhibiting enhanced mechanical properties at 20 wt.% of reinforcement. Furthermore, the tensile and impact strength of PLA composites would be enhanced by 1.5 times if the fabrication is carried out by torque rheometer [64]. PLA is modified with various stabilizers and compatibilizers, as shown in Figure 9. On adding sorbitol and glycerol in sugar beet pulp-reinforced PLA composites [65], the tensile modulus was enhanced by a marginal factor of 5% at 40 wt.% of reinforcement, but the bending strength and storage modulus decreased drastically. Reinforcing carbon fiber in the PLA matrix can increase the tensile strength up to two times regardless of the compatibilizer and stabilizers [66]. However, the biodegradability of the composites was enhanced with the addition of 20 wt.% of chitosan in the mix of carbon fibers and PLA. Bamboo fiber-reinforced PLA composites were prepared via injection molding, and it was reported that bamboo fiber degraded the dynamic properties of PLA due to the weak bonding between bamboo and PLA [67]. Nevertheless, the tan δ of the PLA–bamboo composites experienced a marginal increase compared to that of the pure PLA. Kenaf fibers and rice husk fillers can improve the flexural modulus of PLA, but flexural and impact strength decreases [68]. The drop in the mechanical properties of PLA is significant in rice husk fillers–PLA composites due to the inability of rice husk fillers to make a strong bond with the PLA matrix. Similar observations were obtained for olive husk filler–PLA composites [69], where both tensile strength and elongation at the break decrease due to the poor dispersion of reinforcement in the matrix. So far, reinforcing biofillers in PLA at different loading conditions have not produced any fruitful results for mechanical performance. With an intent to achieve higher mechanical properties that could compete with the strength of synthetic fibers’ composites, flax fibers in the form of a bidirectional mat were reinforced in PLA [70]. Results revealed that absorption energy and elongation at the break were increased by 20% and 32%, respectively. The composites prepared also showed improved ductility due to the reinforcing effect of the bidirectional flax fibers’ mat. Encouraging mechanical properties were also obtained after reinforcing kenaf fibers in the PLA matrix, fabricated by a melt blending technique [71]. Both tensile strength and tensile modulus of the composites increased up to 30% of the fibers’ weightage. The enhancement was attributed to the triacetin, which provided rigidity to the PLA and bound the matrix and fibers together for an improved interfacial bonding. Improper wetting of the kenaf fiber due to the insufficient matrix was the reason behind the reduced mechanical properties of the composites at fibers’ higher reinforcement. Polylactic acid has been modified with various plasticizers to achieve required plasticizing. The substitution of triacetin with 10% polybutylene adipate terephthalate (PBAT) (a plasticizer) in kenaf fibers-reinforced PLA composites can yield the tensile strength of 115 MPa, which is more than twice the value of tensile strength of pure PLA [72]. In another instance, the addition of 20 wt.% PBAT in waste paper-reinforced PLA composites enhanced the impact strength of the composites by 291% of the original value of the PLA [73]. However, the flexural strength and flexural modulus decreased by 17.8% and 26.4%, respectively. Banana fiber-reinforced PLA composites were prepared by a melt mixing process, and it was found that the tensile and flexural strength of the composites was increased significantly, at 40 wt.% of the banana fiber loading [74]. The incorporation of hemp fiber in PLA enhanced the flexural strength, flexural modulus, and impact strength by a factor of 60%, 90%, and 68%, respectively, as compared to pure PLA [75]. Okra fibers enhanced the stiffness of PLA at a reinforcement of 30 wt.% due to the improved interfacial bonding between okra fibers and the PLA [76]. A linear increase in the value of the tensile strength of PLA can be attained upon reinforcing with basalt fibers ranging from 5 wt.% to 30 wt.% [77]. The inclusion of coir fibers (extracted from the outer husk of the coconut) in PLA improved the impact strength at 30 wt.% of reinforcement [78,79], fabricated via a twin-screw extruder and injection molding method. However, a significant decrease was observed in tensile strength and elongation at the break of the composites. An excellent improvement in the mechanical properties of the composites was achieved by incorporating bamboo stick tied by a nod in the PLA [80] due to the hindrance offered by the nod to the movement of resin. In general, PLA has a very low crystallinity of approximately 10%, which is the reason behind its comparatively low mechanical strength, which can be improved by adding 5% benzylated treated rice straw in a solution of PLA and acetylation compound [81,82]. A blending of PLA with a copolymer has frequently been utilized to enhance its chemical bonding with natural fibers/fillers. A blend of polymers containing polycaprolactone (PC), polybutylene succinate (PBS), natural rubber (NR), PLA, and PBAT reinforced with grass fibers increased in tensile strength and modulus, but impact strength decreased [83], as shown in Table 4. The formation of a cross-link polymer due to the merger of a copolymer entangles the fibers, leading to a strong bond with the resin.

Curcumin in PLA decreases the tensile and flexural strength, whereas the addition of chitin brings a severe drop in the tensile and flexural modulus of the PLA [86,104]. Nevertheless, chitin enhances the water-repellent characteristics of the composites due to its hygroscopic characteristics. Similarly, reinforcement of lignin fillers’ extract of orange peel in PLA reduces its tensile strength and modulus [105,106], but thermal stability improves because of the improvement in the melting temperature of the composites. In view of fabricating an economically viable composite, numerous types of wood fillers, such as oak, pine, sprue, etc., were reinforced in PLA. However, the outcomes were strictly futile as the mechanical strength deteriorated and the crystallinity, thermal stability, and energy absorption capacity worsened [93,107,108] due to the brittleness imparted by the wood flour and the weak interfacial bonding between the wood flour particles and PLA resin. Chances of improvement in the mechanical strength of the PLA are marginal only if the wood flour is subjected to acetylation, silane treatment, or any other chemical treatment. The ongoing discussion of fibers’ and fillers’ reinforcement effect on PLA properties can be concluded as fiber imparts greater strength than fillers [109,110] but this does not exclude it from the league of PLA-based composites. Various other ways have evolved for its reinforcement in PLA. Hybridization of filler with fibers is one of them. The tensile strength of the composites increases from 34.84 MPa to 52.75 MPa by mixing ash in lignin fiber-reinforced PLA composites [84,91,111]. The improvement in the tensile strength was attributed to the acetylation compound added in the mixture of fiber and matrix.

Moreover, the tensile and flexural strength increased by 28% and 31%, respectively [112], when the carnauba wax, as the filler, was added in the cellulose fiber-reinforced PLA composites. The carnauba wax in the composites acts as a plasticizer, forming a bridge between the fiber and matrix. The flexural modulus is enhanced by 56% when cotton linters are hybridized with maple fibers, at a 50% reinforcement [113]. The reinforcement of two fibers in PLA was also carried out in the last few years to examine the mechanical properties. Silane-treated banana fiber and sisal fiber-reinforced PLA hybrid composites yielded improved tensile and flexural strength [95], whereas hybrid composites containing empty fruit branch and kenaf fiber yielded better tensile modulus compared to neat PLA [96]. PLA shows greater improvement in strength when reinforced with fibers as compared to fillers, but the ease of manufacturing in a melt blender, injection molding, etc. can be easily achieved with filler reinforcement. As far as the strength of PLA is concerned, it can be increased up to two fold by mixing aluminum triflate at a temperature of 1700 °C. However, rice husk filler degraded the strength of PLA due to a lack of intermechanical bonding. This shows that a filler of metals such as AL, Ti, and their alloys has a greater potential to act as a reinforcing material in PLA composites compared to natural fillers. Table 4 shows the effects of different types of fiber on PLA strength by using different techniques. Although every fiber has its own influence on PLA, kenaf, okra, and banana fibers impart greater strength.

## 8. Effect of Nanofillers on the Properties of PLA Nanocomposites

Nanocomposites are a special class of composites in which a filler of particle size in the nanometer range is reinforced in the polymer matrix. Nanofillers have a vast surface area, due to which a small amount of nanofiller reinforcement is enough to bring a significant change in the properties of the polymer. The literature revealed that 0.5% to 8% nanofiller reinforcement is sufficient to strengthen the polymer mechanically and thermally [114]. Broadly, nanocomposites are classified into three groups, viz., (1) layered nanofillers’ nanocomposites, (2) whiskers’ nanocomposites, and (3) nanoparticles filled nanocomposites. Layered nanofillers are one-dimensional nanomaterials with a thickness of 1 nanometer (nm), for example, sheets of graphene, silicates, and clay, while whiskers are cylindrical in shape, less than 100 nm in diameter, such as carbon nanotubes. With an average diameter of 100 nm and a three-dimensional shape, particles such as metal oxides, silica particles, and polyhedral oligomeric silsesquioxane (POSS) come under the category of nanoparticles [115]. Several types of nanofillers, such as carbon nanotubes (single and multi-wall), silicates’ compounds, hydroxyapatite, aluminum hydroxide, many organic and inorganic fillers, etc., have been reinforced in PLA in recent decades. The findings reported for the PLA-based nanocomposites are exciting and commendable. For instance, when CaCO_3_ nanofiller was reinforced with PLA via a twin-screw extruder, the elastic modulus increased by a factor of 52% but the yield strength and elongation of the break decreased by 19% and 12%, respectively, compared to neat PLA [116]. The bond formation between the CaCO_3_ and lactide compound increased the brittleness of composites, resulting in the reduction in the properties. The addition of plasticizers such as tributyl citrate and polyethylene glycol in PLA can transform the structure from brittle to ductile [117]. CaCO_3_ is a low-cost nanofiller, and it is usually added to bring down the cost of the composite. Composites prepared from CaCO_3_-filled PLA result in agglomeration of nanoparticles due to a high surface energy. The issue of agglomeration can be resolved by modifying the surface of CaCO_3_ by using silane, titanates, zirconates, etc. as a modifier [118,119]. The changes brought by the incorporation of CaCO_3_ in PLA other than mechanical changes include the crystallinity enhancement at elevated temperature because of the heterogeneous nucleation at the matrix of the PLA [120]. However, the CaCO_3_ nanofiller does not contribute much to the property enhancement of the PLA due to the compatibility issue [121], which can be fixed to some level by the incorporation of a chemically treated silica nanofiller in the CaCO_3-_filled PLA nanocomposites, leading to significant improvement in the notched impact strength and elongation at the break. Apart from reinforcing the nanofiller, several factors, such as shape and size, method of preparation, curing time, dispersion technique, etc., play a crucial part in deciding the final property of the PLA nanocomposites.

The incorporation of a silica nanofiller, nanoclay, and starch in the PLA showed similar effects as CaCO_3_ due to the delamination occurring at the filler and matrix interface [122]. Changing the method of fabrication can lead to encouraging results concerning the properties’ enhancement of PLA. Composites of silica nanofiller-reinforced PLA prepared by melt blending showed enhanced mechanical properties and improved flexibility [123]. However, dynamic mechanical analysis of the composites showed no improvement in the viscoelastic behavior and glass transition temperature. Silica is a hard particle and is reinforced to increase the strength, adhesiveness, and abrasive property of the polymer. Polylactic acid becomes more flexible when silica nanofiller is added to it [124], but the strength decreases due to the non-uniform dispersion of silica nanoparticles. To achieve uniform dispersion, grafting is performed on silica nanoparticles by oligomer L lactic acid [125], which modifies the surface and improves the interfacial adhesion between silica nanoflour and PLA, resulting in higher tensile strength and toughness of composites as compared to pristine PLA. Moreover, the tensile strength and elongation at the break increased drastically when trimethyl hexamethylene diisocyanate (TMDI) or 2-methacryloyloxyethyl isocyanate (MOI) was added to the silica nanofiller–PLA composites [126,127]. In addition, silica nanofillers can improve the thermal stability of PLA due to the entanglement of silica nanofiller in the polymeric chain of PLA [128], which results in the formation of complex bonds, enhancing the melting point.

Nanoclay is the compound of silicates and minerals obtained naturally in various forms such as hectorite, kaolinite, bentonite, and montmorillonite. It is cheap and can make a strong bond with the polymer to make the composites mechanically and thermally robust. Reinforcing nanoclay in PLA is a novel idea to implement new findings in the sphere of nanocomposites. For instance, PLA–nanoclay nanocomposites prepared by melt blending and melt intercalation processes exhibited extremely high ductility, improving by 208% compared to neat PLA [129]. The enhancement in ductility is due to the modification of nanoclay with ammonium chloride, causing uniform dispersion in the matrix of the PLA. The clay also imparts compactness in the PLA, resulting in rigid structure formation, improving the mechanical strength.

On the other hand, nanocomposites containing nanoclay prepared by intercalation are more dispersed in layer form in between the layers of the PLA, giving rise to shear banding of multilayers [130], resulting in a large deformation. Moreover, the layered structure of the nanoclay–PLA transforms into exfoliation upon treating the nanoclay with a silane agent, affecting the crystallinity of the PLA [131] due to the drop in nucleation growth at the interface of the composites’ structure. In this context, a conclusion can be made that layered nanocomposites with layered structures exhibit high crystallinity compared to an exfoliated structure. However, due to the low rate of nucleation in an exfoliated structure, the grains’ form is spherical, having a low permeability of gas [132].

Several investigators have fabricated nanocomposites by reinforcing titanium dioxide (TiO_2_) in the PLA matrix. TiO_2_ particles have a very large surface area and are usually reinforced in polymers to infuse photocatalytic and magnetic properties [133]. In addition, toughness, photodegradability, and polymer crystallization are enhanced by the inclusion of TiO_2_ in polymers [134,135,136]. Nanocomposites of TiO_2_ (in nanowire form) and PLA prepared by in situ melt polycondensation showed improved thermal stability and high Tg [137] because of covalent bonding, interfacial bonding adhesion, and homogeneous distribution between the TiO_2_ and PLA. Grafted and polymerized TiO_2-_reinforced PLA nanocomposites, fabricated by a melt blending process, gave high mechanical strength compared to pristine PLA; both mechanical and thermal properties will be enhanced if the nanocomposites are prepared by in situ polymerization [138,139]. Polylactic acid is less susceptible to photodegradation while TiO_2_ nanoparticles have relatively high photodegradability; hence, TiO_2_ nanoparticles can improve the photodegradability of PLA. Apart from that, TiO_2_ substrate-filled PLA nanocomposites can be used as antimicrobial treatment material of biological cells [140] due to the photocatalytic behavior of TiO_2_.

An inorganic compound, known as zinc oxide (ZnO), is regarded as a vital nanofiller in the fabrication of nanocomposites for the property enhancement of polymers due to its good hydrophobicity, decent antibacterial property, and excellent ultraviolet (UV) absorption characteristics [141,142,143,144]. For example, the addition of ZnO in PLA using the melt blending process decreases the mechanical strength of the nanocomposites, which occurs because of the agglomeration of ZnO nanoparticles at a particular place. The strong van der Waals wall force of attraction among the ZnO particles is the major reason for the agglomeration. However, treating ZnO with stearates or fatty amides can modify the particle surface, reducing the van der Waals force of attraction, resulting in enhanced dispersion and improved strength [145]. Very high thermomechanical properties of nanocomposites can also be obtained for tri ethoxy caprylyl silane-treated ZnO nanoparticles-filled PLA composites [146]. It has also been observed that ZnO nanoparticles obstruct PLA crystallization by playing the role of an anti-nucleating agent, which lowers the T_g_ of nanocomposites [147]. The sign of early degradation of PLA can also be achieved by adding ZnO nanofillers at the melt processing temperature [148]. The change in the PLA properties by the addition of typical nanofillers is illustrated in Table 5.

Nanofillers composed of carbon black and graphite, generally known as carbon nanotube (CNT), can bring fruitful changes in polymer properties. The polymer can be made mechanically strong, thermally enhanced, and electrically active with the reinforcement of CNT nanofillers [156,157,158]. A CNT is a cylindrical nanostructure and acts as a surface functionalization in a nanocomposite, which aids in easy solubility and uniform dispersion [159]. In situ polymerization, melt blending, and solvent evaporation are some of the most common methods by which PLA and CNT blends are made. Nanocomposites containing CNT and PLA improve mechanical strength and enhance the fire-retardant properties of the nanocomposites [160]. The structure of PLA becomes highly crystallized by incorporating CNT due to the formation of covalent bonds in the nanocomposites. Chemically oxidized CNT-filled PLA nanocomposites prepared via melt blending techniques exhibit better mechanical strength and thermal resistance than pure PLA [161]. The incorporation of graphite and organically modified montmorillonite (OMMT) at a melt processing temperature transforms the PLA structure, with excellent thermal characteristics, high stability, improved crystallinity, and an enhanced Young’s modulus [162,163,164]. The property enhancement in PLA by a carbonaceous nanofiller is mainly attributed to two primary mechanisms, viz., intercalation, which contributed to strength, and nucleation, enhancing crystallinity. However, the industrial application of carbonaceous nanofiller–PLA composites is limited because of the high input and processing costs.

Incorporating glass nanofillers has also brought physical, mechanical, and thermal changes in PLA [165]. Flexural modulus, flexural and impact strength, and Young’s modulus of PLA are increased by adding a glass nanofiller having a shape of a microsphere, but the elongation at the break decreases [166]. Glass nanofillers can regulate the surface energy of PLA, provided the composites are added with a soluble solution of calcium phosphate Ca_3_(PO_4_)_2_. The mixing of a Ca_3_(PO_4_)_2_ soluble solution forms precipitation at the surface of the PLA, which accelerates the biodegradation rate of the composites. The melting temperature, heat capacity, and glass transition temperature of PLA are also enhanced by the glass nanofiller [167].

Polylactic acid-based nanocomposites containing alumina as a nanofiller are very useful in clinical applications such as maxillofacial reconstruction and tissue growth [168]. Since it belongs to the ceramics’ class, alumina possesses good bio-inert characteristics, outstanding corrosion resistance, and very high strength. Alumina makes excellent bonding with the PLA due to hydrogen bonding and polar coupling and enhances its thermal conductivity. Moreover, alumina nanofiller in PLA makes it mechanically robust and provides rigidness [169]. A wide range of dental and orthopedic materials is made of alumina nanofiller–PLA nanocomposites due to the presence of an ester group in PLA, which enables a strong bond with alumina [170]. A coating of alumina on PLA is also used for biomedical equipment.

Nanofillers made from ferrous oxides are very advantageous when used as reinforcing agents in polymers. Their nanocomposites are viable in various applications such as gas sensors, lubricants, flocculants, sorbents, catalysts, pigments, etc. [171,172,173]. Electronic and magnetic devices such as data storage devices, xerography ink, recording tape, and magnetic resonance imaging can also be made from ferrous oxides’ nanocomposites [174,175,176]. Ferrous oxides are abundant in the Earth’s crust, and their nanofillers can be easily made by a simple technique known as co-precipitation in the presence of suitable surfactants [177]. Ferrous nanoparticles experience inductive heating under the influence of magnetic fields within the nanocomposites when subjected to heat. This behavioral change of ferrous oxide nanoparticles is valuable in the transition of shape memory devices [178,179]. These ferrous oxides’ nanocomposites are prepared by the method of precipitation and exhibit strong adhesion with the PLA via hydrogen bonding. Extensive applications of nanofilms of ferrous oxide and PLA can also be seen in the biomedical industry [180].

**Figure 10:**
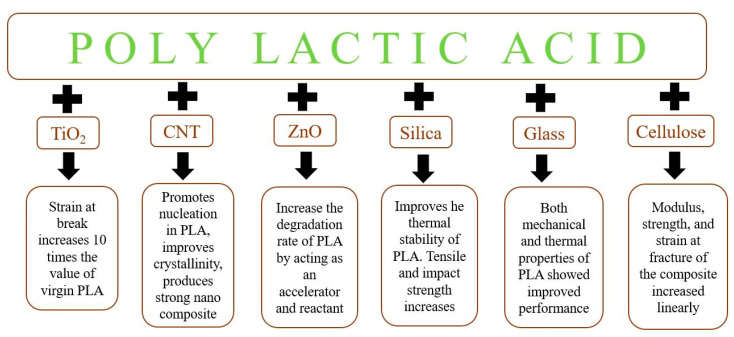
Summarized effect of some typical nanofiller on the properties of PLA.

Fully biodegradable nanocomposites can be made by reinforcing naturally occurring biofibers at the nanoscale in PLA. Cellulose and lignin are the two biofibers that can bring significant changes in the properties of PLA. Nanocomposites prepared via a melt screw extruder containing short cellulose microfibrils and PLA in the presence of a plasticizer exhibited improved mechanical strength compared to pure PLA [181]. Organic solvents, such as esters, ethers, ketones, etc., can be mixed in the nanocomposites to disperse the cellulose microfibrils uniformly in the matrix of the PLA [182,183]. Organic solvent decreases the hydrogen bonding between the cellulose–cellulose microfibrils and allows easy fiber separation [184], resulting in homogenously dispersed nanocomposites. Enhanced mechanical and thermal properties of cellulose–PLA nanocomposites prepared via the stacking process can also be achieved by the treatment of fiber [185]. The cell wall of plants is composed of a large number of aromatic compounds called lignins. High-quality lignin can be extracted from almond shells by the organosolv method. The treatment of lignin with acetylation results in an improvement in strength and stiffness [186]. Nanocomposites based on unmodified lignin and PLA yielded improved thermal and mechanical properties [187]. The summarized effect of a nanofiller on the properties of PLA is shown in Figure 10. From the literature, it was revealed that PLA shows greater improvement in strength when reinforced with fibers as compared to fillers. Strength can be increased up to two fold by mixing aluminum triflate. However, rice husk filler degraded the strength of PLA due to a lack of intermechanical bonding.

## 9. Industrial Scope of PLA

**Figure 11:**
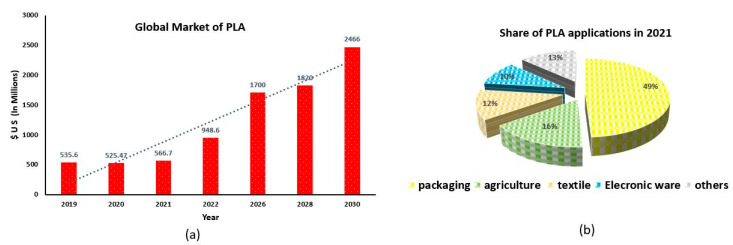
Showing (**a**) forecasted trend of global market size of PLA and (**b**) weightage of various PLA applications in 2021 [214].

As of 2021, the market capital of bioplastic was estimated to be USD 7.6 billion, which accounts for only 5% of the total plastic industry; however, an appreciable growth rate of 30% was reported for the same in the last few years [188,189,190,191,192,193,194]. Irrespective of the industry, the coming years will find a greater dependency on sustainable materials solely made of bioplastics, considering key factors such as the intensification of greenhouse gases, accumulation of waste plastic, and high energy demand in the processing of plastics [195,196,197,198,199,200,201,202]. Being considered as an eco-friendly plastic, PLA is an appropriate material for agriculture products such as bags for the storage of agriculture products, mulch films, paper coating for packaging, and bags for composting, fertilizers, and pesticides’ release systems [203,204,205,206,207,208,209,210,211,212,213]. As shown in Figure 11, the current size and forecasted growth of the market size of PLA is very impressive and may increase at a greater pace due to the strict action taken by the various European and American countries on single-use plastic [214]. PLA has a huge scope in fiber manufacturing. It can be melt-spun and drawn to crystallize stress and can be extruded or molded for the production of fiber. PLA-based fibers are utilized in quilts and cushions as a fiber fill, as a continuous filament form in carpet, as filament and spun yarns in apparel, and as various nonwoven and biocompatible fibers such as self-crimp and binders [215]. Particularly in nonwoven form, PLA finds its application in padding, reusable clothes, canopies, and diapers. Generally, fibers are subjected to thermal and mechanical loads and, therefore, require high heat resistance and stiffness. High crystalline fiber has the ability to perform in critical conditions, as stated above; thus, meso lactic compound (8–20%) is added to PLA, which makes cross-linking chains to increase its adhesion with other fibers [216,217,218,219,220,221]. Earlier, the melt spinning process used polyethylene terephthalate (PET) for fiber spinning; but, because of the biodegradability and impressive mechanical properties of PLA, a huge swing from PET to PLA has been observed in the industry. It is soft in feel, glossy in appearance, and shows outstanding resistance against stains [222]. Products such as disposable coffee and tea cups and packaging material for mustard oil and makeup are now extensively produced from PLA. In addition, the low smoke generation after the burning of PLA, high resistance against ultraviolet radiation, and good wicking ability makes it a suitable material for the dye and textile industries [223,224,225,226,227,228].

PLA has become one of the most valuable polymers in film processing. Films made from PLA exhibit remarkable twist retention and dead folding, characteristics suitable for packaging electronics and mechanical goods [229,230,231,232,233,234]. Fresh products whose quality does not degrade by the presence of oxygen within a certain period of time can also be packed in PLA films. Thermoforming of PLA with sugar, beet, corn, etc. is being used to make packaging containers for retailing fruits, vegetables, and dry fruits [235,236,237,238,239,240,241]. Walmart is the world’s largest retailer, selling its products in PLA containers such as cups for drinking, plates, laminated films for vegetables, cutlery, lids, and straws. The chemical, physical, and mechanical properties of PLA are tailored in accordance with the required temperature and humidity for the retailing of the product, since the latter can be in liquid, solid, or semi-solid form.

The complete shift from synthetic polymers to bioplastics, particularly PLA, has a long way to go due to constraints linked to PLA such as a high manufacturing cost, lower mechanical strength, and low moisture barrier resistance. The properties’ enhancement of PLA can be achieved by blending with copolymers and incorporating nanoparticles. as previously discussed in the bio- and nanocomposites of PLA [242,243,244,245,246,247,248]. In addition, necessary measures have to be taken, such as restraining moisture diffusion and curbing hydrolytic degradation during the processing, storage, and shipping of PLA-based products while ensuring their biodegradability is maintained.

The major drawback of PLA is its brittleness, which restricts its widespread commercial expansion [249,250,251,252,253,254,255]. Various methods have evolved over time to improve its characteristics, such as improving its hydrophobic properties by coating the PLA, laminating biopolymers via extrusion, the mixing of edible oil to enhance the barrier properties of the packaging material, and the preparation of PLA blends with copolymers for specific purposes [256]. PHA, PCL, and PEG are some typical copolymers that are often used in making PLA blends. To improve the flexibility and toughness of PLA, plasticizing is performed with its own monomer [257]. This plasticized PLA possesses mechanical and physical properties comparable to synthetic polymers, viz., polypropylene, polyvinyl chloride, and polystyrene, thus expanding the scope of PLA as a series of flexible and tough products [258]. However, exceeding the plasticizer content beyond 20% in the composition may lead to the separation of phases in a part or whole of the body. The opportunity of PLA in packaging materials expands further due to the incorporation of nanoparticles [259] since nanoparticles bring exceptional rheological changes in the structure of PLA, resulting in low density, stiffness, thermal and mechanical stability, and recyclable packaging material.

## 10. Conclusions and Future Prospective

The attention received by PLA is not just because of its natural availability and biodegradability but also its tailorability, achieved by incorporating several fillers, fibers, and nanoparticles. Moreover, recent conventions on plastics have influenced several countries to work in the area of replacement of synthetic plastic with bioplastic. Due to the influential of PLA, various natural fibers and fillers can be easily reinforced by using different molding techniques. PLA is not only capable of making a strong bond with synthetic fibers but also makes good interfacial interactions with cellulosic and protein fibers. PLA can be molded into different forms, such as films, nanoparticles, hydrogels, composite panels, microcapsules, and so on, that further widen its scope in the engineering, agriculture, and medical domains. Various blends can be made by incorporating copolymers in PLA for the enhancement of its physical and mechanical characteristics. The blends of PLA and copolymers have enormous capability to act as parallel polymers in various packaging and fiber industries. The processing of PLA has become convenient with the advancement of research and development in biobase products. In the area of agriculture, many products such as fertilizer bags, mulch film, and paper coatings have now been prepared with base material such as PLA. However, the cost of manufacturing is still the crucial subject to be addressed for its commercial viability. Researchers need to look for low-cost substrates and high-performance microorganisms to increase the efficiency of LA production and obtain low-cost, high-quality PLA. In addition, novel copolymers need to be developed that can be used as blending polymers with PLA to reduce its cost and consequently improve its strength. Nanoparticles, such as TiO_2_, MgO, ZnO, etc., have the capability to give significant strength to the PLA that can be used in a hybrid form with natural fibers to provide stability to the PLA.

## Figures and Tables

**Table 1:** Typically known biopolymers [10,11,12].

Synthesized Polymers	Produced by Microbial Fermentation	Chemically Modified
Polycaprolactone (PCL)	Polylactic acid (PLA)	Lignocellulose (straw, wood)
Aliphatic co-polyesters (PBSA)	Polyhydroxyalkanoates (PHA)	Lipids and proteins (gelatin, soybean, gluten, collagen, whey)
Polyglycolic acid (PGA)	Poly(3-hydroxybutyric acid-co-3-hydroxyvaleric acid) (PHBV)	Starch (maize, wheat, potato, thermoplastics)
Aromatic co-polyesters (PBAT)	Polyhydroxy butyrate (PHB)	Others (chitosan, chitin, pectin, gum)

**Table 2:** Properties’ comparison of PLA with polymers [30].

Properties	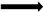	Tensile Modulus (GPa)	Yield Strength (MPa)	Flexural Strength (MPa)	Elongation (%)
Polymer					
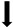					
Polylactic acid (PLA)		3.2	49	70	2.5
Polyvinyl chloride (PVC)		2.6	35	90	3.0
Polypropylene (PP)		1.4	35	49	10
Polystyrene (PS)		3.4	49	80	2.5
Nylon		2.9	71	95	5

**Table 3:** Physical properties of polylactic acid [36].

Property	Values
Specific Gravity	1–1.5
Surface Energy (dynes)	36–40
Melting Temperature (°C)	140–210
Molecular Weight (Daltons)	Approx. 1.6 × 10^5^
Melt Flow Index (g/10 min)	4–22
Crystallinity (%)	5–35
Glass Transition Temperature (°C)	50–75
Solubility Parameters (J^0.5^/cm^1.5^)	21

**Table 4:** Mechanical properties of PLA composites.

Composition	Preparation Technique	Tensile Strength	Flexural Strength	Impact Strength	Modulus (GPa)	Strain (%)	References
PLA-5% Lignin	Twin-screw micro-compounder	48.39 MPa	37 MPa	22.8 KJ/m^2^	1.9 GPa	2.4	[84,85]
PLA-63% starch-24% cellulose-2.9% carnauba wax	Blending, mixing followed by compression molding	3.27 MPa	296.87 KPa	1.9 J	482.93 MPa	0.77	[86,87,88]
70% PLA-20% PBAT-10% office waste paper	Injection molding	49 MPa	73 MPa	15.2 KJ/m^2^	2.9 GPa	3.6	[73,89]
PLA-30% rice straw	Solvent casting	22.27 MPa	26 MPa	30 J	2.59 GPa	1.63	[82,90]
PLA-30% kraft lignin	Extrusion	25.3 MPa	68 MPa	13.2 MPa	1.9 GPa	1.4	[91,92]
PLA-50% pine wood flour	Counter rotating twin-screw micro extruder	66.2 MPa	98 MPa	6 KJ/m^2^	5.4 GPa	1.6	[93,94]
PLA-30% banana–sisal fiber	Injection molding	79 MPa	125 MPa	47.8 KJ/m^2^	4.1 GPa	1.1	[95]
PLA-8% oil seed fillers	Co-rotating twin-screw extruder	62.6 MPa	--	--	1.43 GPa	7.8	[62]
PLA-10% sugar beet pulp	Extrusion and injection molding	38 MPa	--	--	2.2 GPa	3.1	[65]
PLA-30% okra fiber	Co-rotating twin-screw micro extruder	58.4 MPa	--	--	4.6 GPa	1.9	[76]
PLA-60% EFB	Brabender mixer and hot press machine	12.4 MPa	9 MPa	122 J/m	431 MPa	3.3	[96]
PLA-60% kenaf	Brabender mixer and hot press machine	5.2 MPa	28 MPa	67 J/m	321 MPa	4.4	[96]
PLA-30% kenaf bast fiber	Brabender internal mixer and hot press mold	32 MPa	40.5 MPa	12.3 J/m	4.3 GPa	7	[71]
PLA-10% coir fiber	Twin-screw extruder	57.9	107.1	3.08 KJ/m^2^	4.0 GPa	3.7	[78,97]
PLA-40% banana fiber	Counter-rotating internal mixer	78.6 MPa	65.4 MPa	17.1 J/m	7.2 GPa	0.24	[74]
PLA-10% kenaf fiber	Counter-rotating internal mixer	37 MPa	40.5 MPa	196 J/m	4.8 GPa	1.26	[72,98,99]
PLA-hemp fiber	Twin-screw extruder	72.1 MPa	96.5 MPa	2.8 J/m^2^	2.4 GPa	5.6	[75]
PLA-50% jute fiber	Compression molding	32.3 MPa	41.8 MPa	3.5 J	2.11 GPa	2.2	[100]
PLA-50% flax fiber	Compression molding	151 MPa	215 MPA	19.5 KJ/m^2^	18.5 GPa	8.3	[101]
PLA-30% ramie fiber	Compression moulding	53 MPa	104 MPa	9.8 KJ/m^2^	4.3 GPa	3.2	[102,103]

**Table 5:** Influence of nanoparticles on the properties of PLA [146,149,150,151,152,153,154,155].

Nanoparticle Added in PLA	Change in the Properties of PLA			
	Tensile Strength	Young’s Modulus	Melting Point	Crystallization Temperature
1% TiO_2_	2.8%	4.3%	0.2%	5.9%
1% HNT	1.58%	5.28%	−0.68%	-
1% ZnO-Cu-Ag	−30.15%	−22.5%	0.6%	−16.8%
1% ZnO	−2.3%	7.4%	1.1%	2.7%
1% MgO	16.8%	27.5%	−0.5%	4.6%
1% GO-ZnO	20.5%	-	-	-
1% ZiF 8 MOF	9.6%	44.7%	-	-

## Data Availability

No data were used to support this study.
